# Lateral Wall Failure During Implant Osteotomy Preparation in a Standardized Narrow-Ridge Model: Osseodensification Versus Conventional Drilling—An Ex Vivo Controlled Study

**DOI:** 10.3390/dj14070414

**Published:** 2026-07-07

**Authors:** Radomir Nikolic, Jelena Vulovic, Milan Bojovic, Gavrilo Ilic, Andrej Kravanja, Dragana Gabric, Zoran Tatic

**Affiliations:** 1 Department of Dentistry, Faculty of Medicine, University of Priština in Kosovska Mitrovica, 38220 Kosovska Mitrovica, Serbia; milan_dent@hotmail.com; 2 School of Dental Medicine, University of Belgrade, 11000 Belgrade, Serbia; 2018.4005@stomf.bg.ac.rs (J.V.); ilicdrgavrilo@gmail.com (G.I.); 3 School of Dental Medicine, University of Zagreb, 10000 Zagreb, Croatia; andrej.kravanja@orto-nova.hr (A.K.); dgabric@sfzg.hr (D.G.); 4 Ministry of Health Reference Center for Dental Implantology, University Hospital Centre Zagreb, 10000 Zagreb, Croatia; 5 Department of Oral Implantology, Military Medical Academy, 11000 Belgrade, Serbia; ztatic09@gmail.com

**Keywords:** osseodensification, implant site preparation, lateral wall failure, trabecular bone, ridge expansion

## Abstract

**Background**: The aim of this ex vivo study was to compare the resistance to lateral wall failure during sequential implant site preparation using osseodensification (OD) burs and conventional rotary drills in a standardized narrow ridge model. The primary outcome was the drill diameter at first lateral wall perforation. Secondary outcomes included paired perforation occurrence at a clinically relevant diameter (4.0 mm) and perforation morphology (length and width). **Methods**: Twenty standardized trabecular bone blocks were prepared in a paired within-specimen design, with each block receiving two osteotomies: one prepared using conventional rotary drills and the other using OD burs. Osteotomy diameter was sequentially increased according to the manufacturers’ protocols until lateral wall perforation occurred. The drill diameter at first perforation was recorded. Perforation morphology (length and width) was quantified using high-resolution extraoral digital scanning and software-based measurements. Statistical analysis was performed using paired non-parametric tests (α = 0.05). **Results**: OD burs significantly delayed lateral wall perforation compared with conventional drills (median 4.30 vs. 4.00 mm; *p* = 0.0000171). At the 4.0 mm drill step, perforation occurred in 16/20 (80%) conventional versus 2/20 (10%) OD preparations (*p* = 0.0013). Perforation length was significantly greater with conventional drilling (*p* = 0.000681), while no significant difference was observed for perforation width (*p* = 0.073). **Conclusions**: Within the limits of this decorticated trabecular ex vivo model, OD increased resistance to lateral wall failure, delayed perforation onset, and reduced perforation length compared with conventional drilling.

## 1. Introduction

Loss of alveolar bone following tooth extraction represents a significant clinical issue in dentistry. After tooth extraction, rapid resorption of the alveolar ridge occurs, with studies showing that ridge width can decrease by up to 50% within the first year (particularly during the first three months) accompanied by a simultaneous loss of bone height [1]. This progressive alveolar bone atrophy can result in insufficient bone volume for dental implant placement, compromising both the aesthetic and functional outcomes of prosthetic rehabilitation [2]. To compensate for the lost bone volume, various augmentation techniques and biomaterials have been developed, with their selection depending on the type of defect and the planned implant therapy. Preservation of lateral wall integrity is clinically important because perforation may compromise implant placement and increase the need for additional regenerative procedures. Contemporary oral surgery employs several approaches aimed at regenerating resorbed alveolar bone by relying on the biological mechanisms of osteogenesis, osteoinduction, and osteoconduction [3].

Osseodensification (OD) is a relatively recent technique in dental implant site preparation, designed to preserve and densify the existing bone, as opposed to conventional osteotomy, which removes bone tissue through drilling. Traditionally, implant osteotomies have been created using a series of progressively larger drills that evacuate bone chips, resulting in osteotomy walls with reduced bone density, particularly in low-density bone types (Type III/IV), and often leading to inadequate primary implant stability [4,5]. Unlike conventional osteotomy, OD utilizes specially engineered burs. The design of OD burs is characterized by a unique cutting edge geometry with a negative rake angle. This geometry allows the burs, when rotating in reverse, to gently abrade and plastically deform bone while simultaneously compacting bone particles into the surrounding trabecular structure. The process preserves native bone volume and enhances the density of the osteotomy site [6]. Additionally, the combined action of hydraulic pressure generated by the irrigation fluid and displaced bone particles contributes to the expansion of the trabecular bone structure. This property is particularly beneficial during procedures such as internal sinus lifts, as it allows for the atraumatic displacement of adjacent structures, including the Schneiderian membrane [7]. An important phenomenon associated with OD is the “spring-back effect”. Following osteotomy preparation, the elastically deformed bone partially recoils, resulting in an osteotomy diameter slightly smaller than that of the bur. This creates a denser bone zone around the implant site, contributing to increased primary stability [8]. OD has demonstrated clinical utility not only in standard immediate implant placement but also in more challenging scenarios, such as insertion into the interradicular septa of molar extraction sockets [9]. Reported advantages of OD over conventional osteotomy techniques include: enhanced primary implant stability, preservation of native bone tissue, facilitated autologous bone grafting, as compacted bone particles remain within the osteotomy, potential for minimally invasive sinus floor elevation, improved bone–implant contact (BIC), promoting better conditions for osseointegration [10]. Given these benefits, OD has attracted attention as a valuable adjunctive technique in contemporary implantology, particularly in cases involving poor bone quality or the need for enhanced implant stability without extensive augmentation procedures. While previous investigations of OD have largely focused on implant stability, bone density, and histological outcomes, limited attention has been given to the biomechanical limits of lateral wall integrity during sequential implant site preparation. In particular, data regarding the drill diameter at which lateral wall failure occurs in standardized narrow ridge models remain scarce.

The aim of this controlled ex vivo study was to compare resistance to lateral wall failure during sequential implant site preparation using OD burs and conventional rotary drills in a standardized narrow ridge model. The primary outcome was the drill diameter at first lateral wall perforation. Secondary outcomes included paired perforation occurrence at a clinically relevant diameter and the morphological characteristics (length and width) of the resulting perforations.

The null hypothesis was that there would be no difference between OD burs and conventional rotary drills in (I) the drill diameter at first lateral wall perforation (primary outcome), (II) the paired occurrence of perforation at a clinically relevant diameter (4.0 mm), and (III) the morphological characteristics (length and width) of the resulting perforations.

## 2. Materials and Methods

**Figure 1:**
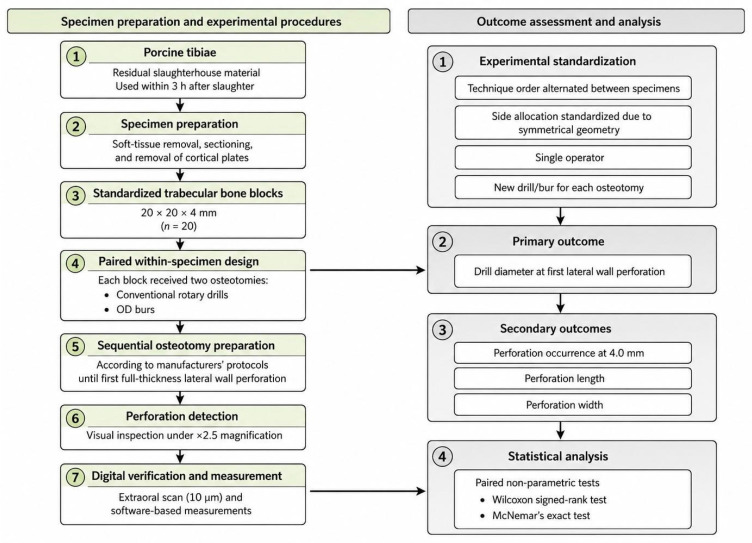
Experimental workflow of specimen preparation, paired osteotomy allocation, perforation detection, digital measurement, and statistical analysis.

This study was designed as a controlled, paired ex vivo experimental model to evaluate resistance to lateral wall failure during implant osteotomy preparation under standardized narrow ridge conditions. The experimental workflow is summarized in Figure 1.

Porcine tibiae were used as bone specimens. The bones were sourced as residual biological material from a local slaughterhouse. No animals were sacrificed specifically for research purposes; therefore, ethical approval was not required under applicable regulations [11]. According to EU Directive 2010/63/EU, the use of tissues derived from animals killed for purposes other than scientific research does not require mandatory ethics committee review [12].

Specimens were obtained within a maximum of 3 h following animal slaughter and were used for experimental procedures on the same day. The bones were transported and temporarily stored in physiological saline solution prior to preparation. No freeze–thaw cycles were applied. During drilling procedures, specimens were kept immersed in water at ambient temperature to prevent dehydration.

The tibiae were cut below the knee joint, and all adhering soft tissues were completely removed. Bone specimens were sectioned using a die cutting machine (Unident^®^, Dentalstall, New Delhi, India) equipped with 0.3 mm thick diamond-coated discs (DFS^®^, Dental-Fabrik Spangenberg GmbH, Riedenburg, Germany) under continuous irrigation with sterile physiological saline solution to minimize thermal damage during sectioning. To standardize the samples, the tibiae were transversely sectioned to remove the medullary portion and cortical plates [13,14], with the specific aim of obtaining trabecular bone blocks measuring 20 mm in length, 20 mm in height, and 4 mm in width. The cortical plates were intentionally removed to isolate trabecular mechanical behavior and eliminate variability related to cortical thickness. Dimensional calibration of the bone blocks was performed using a mechanical thickness caliper (Iwanson caliper, 0–10 mm, Neuhausen, Germany). The model was intentionally designed as a failure-threshold experimental condition rather than a clinical simulation. By selecting a ridge width equivalent to the final osteotomy diameter, the study aimed to evaluate the biomechanical limits of lateral wall resistance under controlled critical conditions.

**Figure 2:**
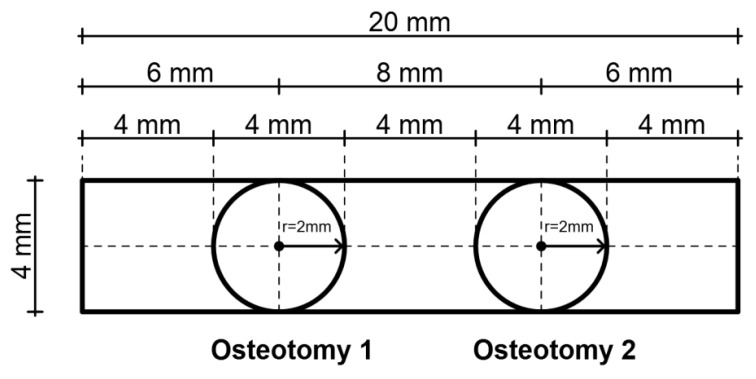
Standardized osteotomy layout. Two centrally aligned osteotomies (Ø4 mm) were created in a 20 mm × 4 mm bone block, positioned 6 mm from each longitudinal border and 8 mm apart (center-to-center), yielding a 4 mm edge-to-edge distance.

Twenty standardized trabecular bone blocks were used in this study. Each bone model was prepared to receive two implant osteotomies: one performed using OD burs and the other using conventional rotary drills (*n* = 20). All specimens were harvested from the same anatomical region of porcine tibiae obtained from animals of comparable commercial body size. A paired within-specimen design was used, with both OD and conventional drilling performed in the same trabecular bone block. Consequently, each specimen served as its own control, reducing the influence of inter-specimen differences in trabecular architecture and bone density on the comparison between drilling techniques. The order of drilling techniques within each block was alternated to reduce potential sequence-related bias. Given that the final osteotomy diameter (4 mm) corresponded exactly to the total width of the trabecular bone block (4 mm), each osteotomy was centrally positioned along the longitudinal axis of the specimen, resulting in tangential contact between the osteotomy walls and the lateral borders of the block, without any additional lateral safety margin. As illustrated in Figure 2, the osteotomy centers were located 6 mm from each longitudinal end of the 20 mm bone block, thereby ensuring symmetrical positioning along its length. Furthermore, the center-to-center distance between the two adjacent osteotomies was standardized at 8 mm, yielding an inter-osteotomy edge-to-edge distance of 4 mm (Figure 2).

Each standardized bone model received two osteotomies in a paired experimental design: one prepared using conventional rotary drills (Neobiotech^®^, Seoul, Republic of Korea) and the other using OD burs (Densah^®^, Versah LLC, Jackson, MI, USA). The sequence of preparation (OD versus conventional drilling) was alternated between specimens to minimize potential sequence-related bias. Because the trabecular blocks were geometrically symmetrical and centrally positioned, side allocation within each block was standardized rather than randomized. Each drill and bur was used only once, and a new instrument was employed for every osteotomy in order to eliminate potential confounding effects related to instrument wear. The geometric configuration of the osteotomies and the inter-site spacing were established in accordance with previously described experimental models of narrow ridge preparation [14] (Figure 2). The symmetrical geometry of the model ensured equivalent boundary conditions for both osteotomy sites. Therefore, no systematic side-related biomechanical advantage was expected, and randomization of side allocation was considered unnecessary.

The bone blocks were placed in custom-made silicone molds fabricated from Zetaplus^®^ (Zhermack S.p.A., Badia Polesine, Italy) and subsequently fixed in a dental paralleling device (Paraskop^®^ M, BEGO, Bremen, Germany) to ensure proper alignment and standardized implant site preparations according to the manufacturers’ protocols (Table 1). For both drilling systems, the osteotomy diameter was progressively increased in a sequential manner (Table 1), and the drill diameter at which lateral wall failure occurred was recorded. All procedures were performed by the same operator, ensuring consistency throughout the study. Drilling procedures were performed using manually applied axial pressure. No device-based measurement or control of insertion force was used. To reduce procedural variability, identical equipment, standardized specimen fixation, a dental paralleling device, and drilling protocols following the manufacturers’ recommendations were employed. Because of the nature of the intervention, the operator performing the osteotomies could not be blinded to the drilling technique. A standardized pecking (pumping) motion was used in accordance with the respective manufacturers’ recommendations. Irrigation was set at 60% of the unit’s maximum flow rate to ensure adequate cooling during osteotomy preparation.

**Figure 3:**
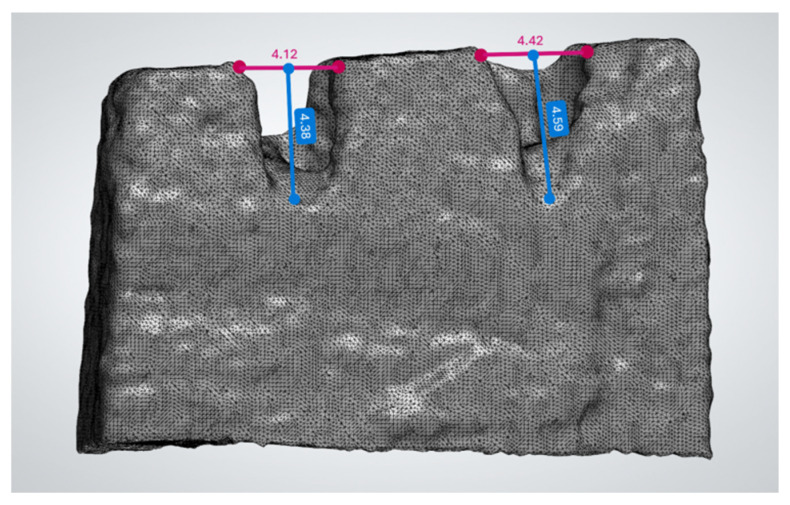
Extraoral laboratory scan of the bone specimen illustrating osteotomy sites and digital measurements of lateral wall perforation width and length; the first osteotomy was prepared using an OD bur with a diameter of 4.3 mm, while the second was prepared using a conventional rotary drill with a diameter of 4.0 mm.

Lateral wall perforation was defined as a full-thickness breach of the lateral border communicating with the osteotomy cavity. Superficial defects, surface irregularities, and incomplete cracks without complete communication with the osteotomy cavity were not classified as perforations. Perforation occurrence was initially identified by direct visual inspection under standardized lighting conditions. Digital scanning was performed only after visual identification of perforation. No additional perforations were detected on digital scans that had not been identified during the initial visual assessment. Agreement regarding perforation presence was complete between examiners, and no discrepancies requiring adjudication occurred. All visual assessments were performed at ×2.5 magnification using surgical loupes (Heine Optotechnik GmbH & Co. KG, Herrsching, Germany) under consistent illumination to ensure reproducibility. Inter-examiner calibration was conducted prior to measurements, and agreement was confirmed before independent assessment. Subsequently, each specimen was subjected to extraoral digital scanning with a high-resolution laboratory scanner (Medit Corp., Seoul, Republic of Korea; 10 µm). The acquired digital models were analyzed in software ver. 3.4.12 (Medit Link^®^, Medit Corp., Seoul, Republic of Korea) to quantify the width and length of lateral wall perforations (Figure 3). All measurements were performed independently by two calibrated examiners who were blinded to the drilling technique. In cases of discrepancy, a consensus value was established following joint re-evaluation of the digital model. Agreement between examiners was confirmed prior to independent measurements. Inter-examiner reliability for perforation width and length measurements was assessed using the intraclass correlation coefficient (ICC). Excellent agreement was observed (ICC = 0.94 for width and 0.96 for length).

Statistical analyses were performed using IBM SPSS Statistics, ver. 22 (IBM Corp., Armonk, NY, USA). Because both osteotomies were prepared within the same trabecular bone specimen, all analyses were paired. The primary outcome, drill diameter at first lateral wall perforation, was analyzed as an ordered paired variable using the two-sided Wilcoxon signed-rank test. The binary secondary outcome was defined as first perforation occurring exactly at the 4.0 mm drill step and was analyzed as a paired binary variable using McNemar’s exact test. Perforation length and width were treated as continuous paired variables. Normality of paired differences for continuous outcomes was assessed using the Shapiro–Wilk test; because normality could not be safely assumed and the sample size was small, paired comparisons were performed using two-sided Wilcoxon signed-rank tests. Results are presented as median [IQR]. Hodges–Lehmann paired differences with 95% confidence intervals were calculated. For Wilcoxon analyses, standardized effect size was calculated as r = |Z|/√N, where N denotes the number of non-zero paired differences for the respective analysis. Statistical significance was set at α = 0.05. No formal adjustment for multiple comparisons was applied because one primary outcome was prespecified and secondary outcomes were interpreted as supportive analyses. The sample size of 20 paired specimens was chosen pragmatically for this ex vivo proof-of-concept model, with the within-specimen paired design expected to reduce between-specimen variability and improve analytical efficiency. Because the study was conceived as an exploratory proof-of-concept investigation, no formal a priori sample size calculation was performed. To facilitate interpretation of the findings, effect sizes and 95% confidence intervals were reported for all principal outcomes.

## 3. Results

A statistically significant difference was observed in the primary outcome, the drill diameter at first lateral wall perforation, between OD burs and conventional rotary drills. Lateral wall perforation occurred in all osteotomies during sequential preparation; no specimen required protocol termination before failure. The median drill diameter at first lateral wall perforation was 4.30 [4.30–4.30] mm for OD burs and 4.00 [4.00–4.00] mm for conventional rotary drills. Two-tailed Wilcoxon signed-rank testing demonstrated a significant difference between techniques (Z = −4.30, *p* = 0.0000171, r = 0.96). All 20 paired specimens showed perforation at a smaller drill diameter with conventional drilling. The Hodges–Lehmann paired difference was 0.30 mm (95% CI 0.30–0.40), indicating later perforation with OD.

**Figure 4:**
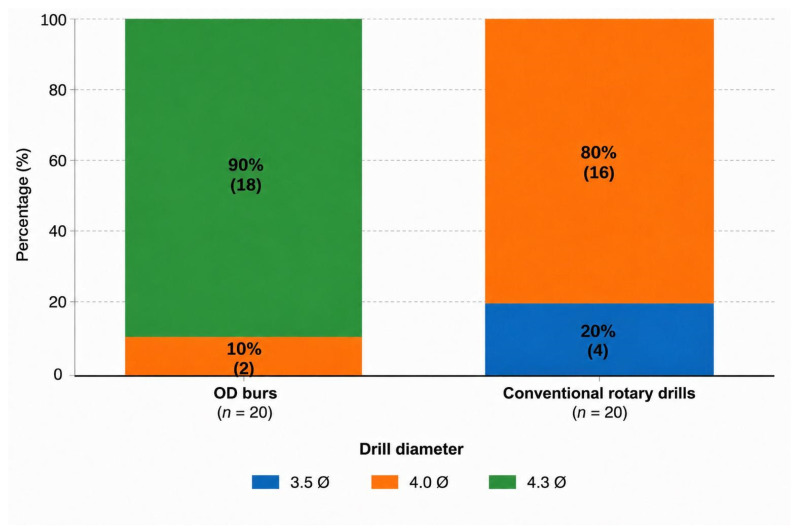
Distribution of lateral wall perforations according to drill diameter for OD burs and conventional rotary drills. Colors indicate drill diameters (3.5 mm, 4.0 mm, and 4.3 mm).

The distribution of lateral wall perforations across sequential drill diameters is presented in Figure 4. In the OD group, perforation occurred at 4.0 mm in 2 specimens (10%) and at 4.3 mm in 18 specimens (90%). In the conventional drilling group, perforation occurred at 3.5 mm in 4 specimens (20%) and at 4.0 mm in 16 specimens (80%).

**Figure 5:**
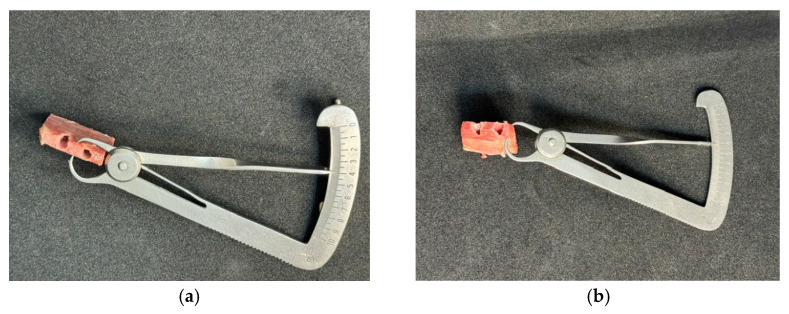
Representative examples of lateral wall perforation patterns. Bone block dimensions were measured using a caliper [mm]. (**a**) Lateral wall perforation following osteotomy preparation with a conventional rotary drill of 4.0 mm; no perforation was observed using an OD bur of the same diameter. (**b**) Lateral wall perforation observed after preparation with a conventional rotary drill of 4.0 mm and with an OD bur at a diameter of 4.3 mm.

To further evaluate paired perforation occurrence at the clinically relevant threshold of 4.0 mm, McNemar’s exact test was performed. At the 4.0 mm drill step, perforation occurred in 16/20 conventional osteotomies (80.0%, 95% CI 56.3–94.3%) and 2/20 OD osteotomies (10.0%, 95% CI 1.2–31.7%). In paired analysis, 16 specimens showed perforation at 4.0 mm with conventional drilling but not with OD, whereas 2 specimens showed the opposite pattern. McNemar’s exact test confirmed a statistically significant difference between techniques (exact *p* = 0.0013). Representative examples of perforation patterns are shown in Figure 5.

Perforation dimensions are summarized in Table 2 as median and interquartile range (IQR). Perforation length was significantly greater following conventional drilling compared with OD burs (3.95 [3.78–4.37] mm vs. 3.52 [3.15–3.71] mm; Z = −3.40, *p* = 0.000681, r = 0.76). The Hodges–Lehmann paired difference was −0.72 mm (95% CI −1.26 to −0.33). In contrast, although perforation width tended to be greater with conventional drills (4.37 [3.35–4.51] mm vs. 3.95 [3.53–4.11] mm), the difference did not reach statistical significance (Z = −1.79, *p* = 0.073, r = 0.40). The Hodges–Lehmann paired difference was −0.39 mm (95% CI −0.64 to 0.15), suggesting a potential trend toward smaller perforation width with OD.

Data are presented as median and interquartile range (IQR). Statistical comparisons were performed using the Wilcoxon signed-rank test. Effect size (r) was calculated as |Z|/√N (N = 20). Statistical significance was set at *p* < 0.05.

## 4. Discussion

Narrow residual alveolar ridges can complicate implant placement and increase the risk of buccal dehiscence, fenestrations, peri-implantitis, and esthetic compromise [15]. To address this challenge, grafting procedures and atraumatic ridge expansion techniques (ARETs) are commonly employed [16]. More recently, OD has emerged as an alternative approach that promotes both ridge expansion and bone densification [8,17,18]. Histomorphometric studies have shown that OD can create lateral and apical zones of mineralized bone, up to 1 mm thick, that support bone regeneration [19,20]. In addition, OD has been associated with increased implant stability quotient (ISQ) values, potentially through the “spring-back” effect, which may contribute to improved secondary stability [21,22].

In the present study, porcine bone was used as the experimental substrate due to its structural and mechanical similarities to human bone, although it is generally considered to have a denser trabecular architecture [23,24]. To evaluate the biomechanical behavior of OD, a standardized 4 mm wide ridge model was used, and osteotomies were prepared until lateral wall perforation occurred. A ridge width of 4 mm was selected because ridges measuring 4–6 mm are classified as narrow (Class III), a clinically challenging condition for implant placement [25]. However, the model was designed as a standardized biomechanical challenge rather than a complete representation of a clinical narrow alveolar ridge. This approach enabled direct assessment of resistance to lateral wall failure and comparison between OD burs and conventional rotary drills under controlled conditions. Perforation morphology was quantitatively assessed using a high-resolution extraoral laboratory scanner (10 µm accuracy).

The present study demonstrated significantly greater resistance to lateral wall failure with OD burs than with conventional rotary drills in a standardized narrow ridge model. At the same drill diameter of 4 mm, osteotomies prepared using OD burs resulted in lateral wall perforations in 10% of cases, whereas conventional drilling led to perforation in 80% of specimens. The large paired effect size (r = 0.96) indicates a strong and consistent difference between techniques. However, this effect should be interpreted within the context of the highly standardized failure-threshold model, which may have amplified the observed effect estimate.

Analysis of perforation morphology revealed distinct patterns between the two drilling techniques. While no statistically significant difference was detected in perforation width, perforation length differed significantly between OD burs and conventional drills, suggesting that OD influences the direction and extent of bone deformation rather than simply reducing the occurrence of perforation. These findings further support the distinct biomechanical response observed with OD under the controlled conditions of the present model.

From a biomechanical perspective, the observed delay in lateral wall perforation with OD may be explained by the distinct bone deformation patterns induced by the two preparation techniques. Conventional drilling removes bone tissue, progressively thinning the lateral wall and potentially facilitating crack propagation as wall thickness decreases. In contrast, OD has been proposed to compact and plastically deform trabecular bone, potentially increasing local bone density and generating a lateral compressive stress field around the osteotomy [6,8]. It may be hypothesized that the formation of a densified peripheral zone contributes to a buttressing effect that enhances the load-bearing capacity of the lateral wall. The shorter perforation length observed with OD may be consistent with the hypothesis that bone compaction limits crack propagation. In trabecular materials, crack propagation and failure pathways may be influenced by local trabecular orientation, connectivity, and regions of lower structural resistance [26]. Therefore, compaction of the peri-osteotomy trabecular network may alter these failure pathways, thereby limiting the longitudinal propagation of the defect. These biomechanical considerations are consistent with the observed quantitative findings, including the delayed perforation threshold and reduced perforation length associated with OD. However, because the underlying microstructural mechanisms were not directly evaluated, these interpretations remain speculative and should be regarded as plausible explanatory hypotheses requiring histological or microstructural validation.

These findings are consistent with previous studies reporting the mechanical and biological advantages of OD compared with conventional drilling techniques [19,20]. A recent systematic review emphasized the importance of buccal bone preservation and peri-implant defect management in compromised ridge situations [27]. Although implant placement was not evaluated in the present study, the delayed onset of lateral wall perforation observed with OD may theoretically facilitate preservation of peri-implant hard tissue architecture. OD has been shown to facilitate ridge expansion and improve bone preservation in narrow ridges, thereby reducing the risk of buccal dehiscence and fenestration while allowing for simultaneous implant placement [28]. Nevertheless, these potential clinical implications require confirmation in future clinical studies. Most previous studies have focused on implant stability and bone density, whereas the present study specifically addresses the biomechanical limits of lateral wall integrity during osteotomy preparation.

Experimental and in vivo studies have demonstrated that OD is particularly effective in low-density cancellous bone (D3 and D4), where it promotes bone compaction, enhances bone-to-implant contact (BIC), and improves primary implant stability [29,30,31,32,33]. These effects have been attributed, in part, to reverse elastic compression of bone [8,20]. In line with these observations, the present study demonstrated greater resistance to lateral wall failure with OD burs compared with conventional rotary drills in a standardized narrow-ridge model. These results further corroborate previous experimental findings indicating that OD may provide biomechanical advantages during osteotomy preparation [19,20]. However, the findings should be interpreted within the limitations of the simplified trabecular ex vivo model used in this study, which was not designed to evaluate implant-related clinical outcomes.

An additional methodological consideration is that the comparison was performed between two complete osteotomy preparation systems rather than isolated drilling variables. The OD and conventional protocols differed not only in rotational direction but also in bur geometry, rotational speed, drilling sequence, and diameter progression, reflecting the manufacturers’ recommended clinical protocols. The higher rotational speed used with OD burs may also have contributed, at least in part, to the observed differences in lateral wall perforation resistance [8,20]. Consequently, the observed differences cannot be attributed exclusively to the OD mechanism itself but may also reflect the combined influence of instrument design and drilling protocol characteristics. In addition, the OD protocol included a greater number of intermediate drilling steps than the conventional protocol. Therefore, the observed differences may have been influenced not only by bone compaction but also by the more gradual enlargement of the osteotomy, which could potentially reduce localized stress concentrations and delay lateral wall failure. Future studies designed to isolate individual variables may further clarify the relative contribution of these factors.

The experimental conditions applied in this study were intentionally demanding, which may have contributed to the pronounced differences observed between the two drilling techniques. Such an approach was chosen to enable evaluation of biomechanical limits under controlled conditions rather than to simulate a clinical drilling protocol. Therefore, the observed perforation thresholds should not be interpreted as direct clinical safety limits for implant site preparation.

Several limitations should be considered when interpreting these findings. The ex vivo model does not account for biological factors such as vascularization, bone remodeling, and healing. In addition, the limited sample size and absence of histological assessment restrict insight into microstructural changes associated with the different preparation techniques. Although the sample size was selected pragmatically, the large effect sizes and narrow confidence intervals for the primary outcome suggest that the study was adequately powered to detect between-technique differences. Another limitation is the absence of quantitative bone density characterization, as bone mineral density measurements, micro-CT, histomorphometric analyses, and Hounsfield-unit equivalent assessments were not performed. Consequently, the structural properties of the trabecular specimens could not be directly quantified or compared with previous studies. Furthermore, the magnitude of the OD effect may vary according to baseline bone density, which could not be assessed in the present study. However, the paired within-specimen design, in which both drilling techniques were evaluated in the same trabecular bone block, reduced the influence of inter-specimen variability.

Another limitation relates to the manual application of drilling forces. Although all osteotomies were performed by a single operator using a standardized protocol, no objective control of axial force, pecking amplitude, or advancement rate was employed. Nevertheless, the single-operator approach, standardized specimen positioning, and paired design helped reduce procedural variability. Future studies should incorporate quantitative bone characterization and force-controlled drilling systems to further improve experimental standardization.

Additionally, the removal of cortical plates and the use of a purely trabecular model may limit extrapolation to clinical ridge conditions. In clinical reality, narrow alveolar ridges usually retain at least a partial cortical component, and therefore the biomechanical behavior observed in the present model cannot be considered fully representative of clinical osteotomy preparation. The absence of the cortical component may have altered stress distribution during the OD protocol, as cortical bone contributes to load transfer under clinical conditions. This may have increased localized trabecular compression, thereby limiting the direct clinical applicability of the findings.

Future studies should incorporate histological and advanced imaging analyses to further characterize bone response and validate the observed perforation patterns. In addition, implant placement was not performed; therefore, no conclusions can be drawn regarding implant-related clinical outcomes.

## 5. Conclusions

Within the limitations of this paired ex vivo trabecular narrow-ridge model, OD delayed first lateral wall perforation and reduced perforation length compared with conventional rotary drilling. Evidence for a between-technique difference in perforation width was insufficient in the present sample. These findings suggest greater resistance to lateral wall failure with OD under controlled failure-threshold conditions. However, because the model does not reproduce the cortical architecture and stress distribution of a clinical narrow alveolar ridge, further studies incorporating cortical bone, including in vivo and clinical investigations, are required before extrapolating these findings to clinical outcomes and safety margins.

## Figures and Tables

**Table 1:** Osteotomy preparation protocols for conventional drilling and OD burs.

Drill System *	Point Drill → Drill Sequence [Ø mm]	Speed [rpm]	Rotation	Osteotomy Depth [mm]
Neobiotech^®^	2.2 → 3.0 → 3.5 → 4.0	800	Clockwise	10
Densah^®^	2.0 → 2.3 → 3.0 → 3.3 → 3.5 → 4.0 → 4.3	1100	Counterclockwise	10

* All implant site preparations were performed using a surgical motor (Woodpecker^®^ Implant Motor, Guilin Woodpecker Medical Instrument Co., Guilin, China) under saline irrigation, with the bone blocks stabilized in a dental paralleling device (Paraskop^®^ M, BEGO, Bremen, Germany) to ensure standardized angulation and reproducibility of drilling orientation. Drilling parameters strictly followed the respective manufacturers’ recommendations for each system. Differences in rotational speed and drill sequence reflect inherent design characteristics of the systems rather than experimental manipulation.

**Table 2:** Comparison of perforation dimensions between OD burs and conventional rotary drills [mm].

	OD Burs	Conventional Drills	Paired Comparison				
Perforation Outcome	Median [IQR]	Median [IQR]	Z	*p* Value	r	HL Difference	95% CI
Width	3.95 [3.53–4.11]	4.37 [3.35–4.51]	−1.79	0.073	0.40	−0.39	−0.64 to 0.15
Length	3.52 [3.15–3.71]	3.95 [3.78–4.37]	−3.40	0.000681	0.76	−0.72	−1.26 to −0.33

Effect size—r.

## Data Availability

The datasets supporting the findings of this study consist of paired measurements of drill diameter at first lateral wall perforation, perforation occurrence at specific drill diameters, and quantitative perforation morphology (length and width). These data are available from the corresponding author upon reasonable request.

## Abbreviations

The following abbreviations are used in this manuscript:

ARETs	Atraumatic ridge expansion techniques
BIC	Bone–implant contact
ISQ	Implant stability quotient
OD	Osseodensification
